# Genetically predicted inflammatory proteins mediate the association between gut microbiota and renal cell carcinoma

**DOI:** 10.1007/s12672-025-01980-y

**Published:** 2025-02-20

**Authors:** Xinyun Zou, Dong Li, Ling Zhang, Jinlan Shen

**Affiliations:** 1 Department of Oncology, The General Hospital of Western Theater Command, Chengdu 610083, China; 2 Department of Laboratory Medicine, The General Hospital of Western Theater Command, Chengdu 610083, China

**Keywords:** Gut microbiota, Inflammatory protein, Renal cell carcinoma, Mendelian randomization

## Abstract

**Background:**

Studies have indicated a potential relationship between gut microbiota and renal cell carcinoma. However, the causal relationship between various types of gut microbiota and renal cell carcinoma, as well as the role of inflammatory protein as mediators, remains unclear.

**Methods:**

This study aimed to identify the relationship between gut microbiota, inflammatory protein, and renal cell cancer through a large-scale genome-wide association study (GWAS) utilizing pooled data. We employed Mendelian randomization (MR) to investigate the causal relationship among these variables. Inverse variance weighting (IVW) was utilized as the primary statistical method. Furthermore, we examined the mediating role of inflammatory protein in the pathway through which gut microbiota influences the development of renal cell cancer.

**Results:**

The analysis revealed 12 positive causal relationships and 15 negative causal relationships between the genetic liability of gut microbiota and renal cell cancer. Furthermore, there were three positive causal relationships and one negative causal relationship between inflammatory proteins and renal cell cancer. There were two axes of relationships in which gut microbiota promote the development of renal cell cancer. through inflammatory proteins acting as mediators.

**Conclusions:**

Gut microbiota and inflammatory protein were causally related to renal cell cancer, and inflammatory protein were intermediary factors in the pathway between gut microbiota and renal cell cancer.

**Supplementary Information:**

The online version contains supplementary material available at 10.1007/s12672-025-01980-y.

## Introduction

Renal cell carcinoma (RCC) is a malignant tumor that originates from renal epithelial cells, accounting for over 90% of all kidney cancer cases [1]. In the United States, approximately 81,610 new cases of kidney cancer are anticipated in 2024, representing 4.1% of all newly diagnosed cancers, with an estimated 14,390 deaths, which account for 2.4% of all cancer-related fatalities [2]. Techniques such as laparoscopic minimally invasive surgery, targeted therapy, immunotherapy, stereotactic body radiotherapy (SBRT), cryoablation, and radiofrequency ablation have provided kidney cancer patients with a wider array of treatment options and improved prognoses [3]. However, early diagnosis of kidney cancer remains challenging due to its high heterogeneity, poor responsiveness to conventional chemotherapy and radiotherapy, frequent resistance to targeted therapies and immunotherapy, as well as significant risks of recurrence and metastasis. Consequently, further research and the development of personalized treatments are urgently required.

The human gut microbiota is a complex community of microorganisms residing in the intestines, primarily composed of bacteria, fungi, viruses, and protozoa. The human gut contains approximately 100 trillion bacteria, encompassing over 1000 different species, with the predominant phyla being Firmicutes and Bacteroidetes [4]. These microorganisms play crucial roles in digestion, metabolism, and immune regulation. Research has demonstrated a complex relationship between the gut microbiota and RCC [5, 6]. Furthermore, the gut microbiota significantly influences the regulation of inflammatory cytokines [7, 8].

Gut microbiota can produce various inflammatory mediators that induce chronic inflammation in the kidneys, thereby increasing the risk of RCC [9, 10]. Consequently, we consider inflammatory cytokines as key mediators in the pathway linking gut microbiota to RCC. Most current research findings are derived from randomized controlled trials, which include observing changes in the gut microbiota in the feces of RCC patients, as well as indirect interventions such as probiotic supplementation or fecal microbiota transplantation aimed at regulating the gut microbiota [11, 12]. However, limitations in objective regulation, such as strain selection, pose challenges for conducting these studies in human populations.

Genome-Wide Association Studies (GWAS) are employed to identify genetic variations associated with specific traits or diseases by scanning the entire genome for single nucleotide polymorphisms (SNPs) linked to these conditions [13, 14]. Mendelian Randomization (MR) is a genetic epidemiology method that utilizes genetic variations as instrumental variables (IVs) to assess causal relationships between exposure factors and diseases [15, 16]. By integrating GWAS data with MR methods, we can more accurately evaluate the causal relationships among gut microbiota, inflammatory cytokines, and RCC.

## Methods

### Study design

**Fig. 1:**
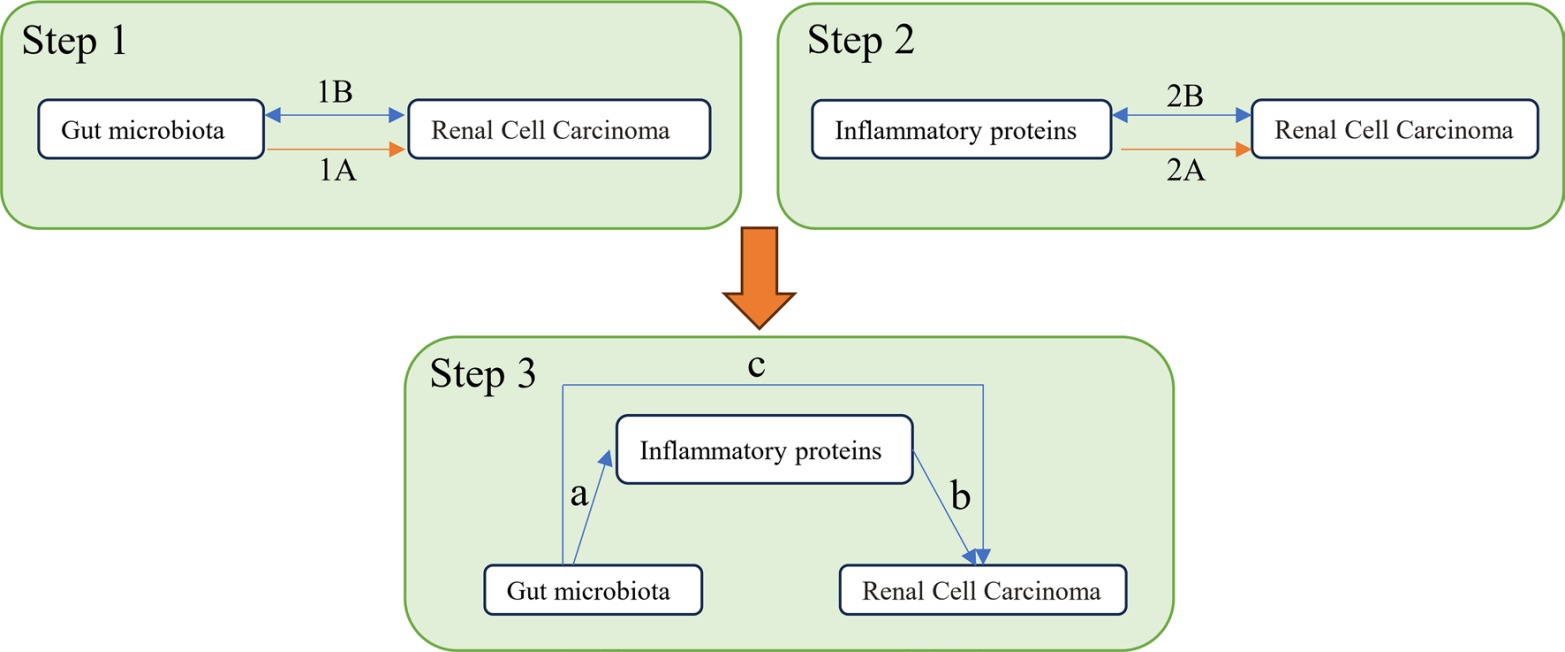
Study overview. Step 1A represents the causal effects of gut microbiota on RCC. Step 1B represents the reverse causal effects of RCC on gut microbiota. Step 2A represents the causal effects of inflammatory proteins on RCC. Step 2B represents the reverse causal effects of RCC on inflammatory proteins. Step 3 represents the mediating analysis of inflammatory proteins in the pathway from the gut microbiota to RCC: path c was the total effect of gut microbiota on RCC; path b was the causal effect of inflammatory proteins on RCC; path a was the causal effect of gut microbiota on inflammatory proteins

This study comprises three parts, as illustrated in Fig. 1: the first part analyzes the causal relationship between 471 gut microbiota species and RCC (Step 1 1A), as well as the reverse causal relationship (Step 1 1B); the second part examines the causal effects of 91 inflammatory proteins on RCC and their reverse causal effects (Step 2); and the final part investigates the mediating effects of inflammatory proteins in the pathway from gut microbiota to RCC (Step 3). In this study, we define SNPs as IVs. The MR analysis is grounded in three core assumptions: (1) Relevance assumption: IVs are significantly associated with the exposure variables; (2) Independence assumption: IVs are independent of confounding factors; and (3) Exclusion restriction assumption: IVs influence the outcome solely through the exposure variables and not via alternative pathways [17].

### Data source

The complete genetic data of gut microbiota are publicly available in the NHGRI-EBI GWAS catalog, with accession numbers GCST90032172 to GCST90032644. This genome-wide association study (GWAS) analyzed the associations between 567 independent SNPs and classifications of gut microbiota, exploring potential links between gut microbiota and various diseases, including colorectal cancer. The GWAS summary data encompasses 471 gut microbiota taxa, which are classified into 11 phyla, 24 orders, 62 families, 146 genera, and 209 species [18].

Similarly, the complete genetic data for inflammatory proteins are also publicly available in the GWAS catalog, with accession numbers GCST90274758 to GCST90274848. This GWAS focused on 91 inflammation-related proteins, which are categorized into several types, primarily including cytokines, chemokines, and other proteins associated with immune responses. The study identified 180 significant primary pQTL signals, comprising 59 cis and 121 trans signals. These findings provide critical insights into the genetic basis of inflammation-related diseases [19].

The GWAS summary data for RCC is derived from the 11th edition of the FinnGen study, a large-scale genomics initiative that has analyzed over 500,000 Finnish biobank samples. This initiative correlates genetic variation with health data to enhance the understanding of disease mechanisms and predispositions. The project is a collaborative effort involving research organizations and biobanks within Finland, as well as international industry partners. We downloaded the genetic data for RCC from the FinnGen study, which includes 3009 cases and 345,118 controls [20]. The download link for the data is: https://storage.googleapis.com/finngen-public-data-r11/summary_stats/finngen_R11_C3_KIDNEY_NOTRENALPELVIS_EXALLC.gz.

The data analyzed in our study are derived from publicly available GWAS summary statistics, and each original GWAS study has received ethical approval; therefore, no new ethical review board approval is required for this study.

### Instrumental variables selection

We selected SNPs that were most significantly associated with gut microbiota and inflammatory proteins, using a threshold of p < 5 × 10^–6^. Subsequently, we excluded SNPs that exhibited linkage disequilibrium (LD) in our analysis. Specifically, the LD for SNPs strongly associated with gut microbiota and inflammatory proteins was required to meet the criteria of r^2^ < 0.001 and a distance greater than 10,000 kb [21]. During the analysis, we ensured that the effects of the SNPs on both exposure and outcome corresponded to the same allele, and we removed palindromic SNPs after matching the results. To assess the strength of the identified IVs, we calculated the explained variance (R^2^) and F-statistic parameters. Generally, SNPs with F-statistic values less than 10 are considered weak instruments [22]. In this study, R^2^ was calculated using the formula

$${\text{R}}^{{2}} \, = \,{2}\, \times \,{\text{EAF}}\, \times \,\left( {{1} - {\text{EAF}}} \right)\, \times \,\beta^{{2}} /\left( {{2}\, \times \,{\text{EAF}}\, \times \,\left( {{1} - {\text{EAF}}} \right)\, \times \,\beta^{{2}} \, + \,{2}\, \times \,{\text{EAF}}\, \times \,\left( {{1} - {\text{EAF}}} \right)\, \times \,{\text{N}}\, \times \,{\text{SE}}^{{2}} } \right),$$

where N represents the sample size, and F is determined by the formula ${\text{F}}\, = \,{\text{R}}^{{2}} \, \times \,\left( {{\text{N}} - {2}} \right)/\left( {{1} - {\text{R}}^{{2}} } \right)$[23].

### MR analysis

We employed a two-sample MR analysis method to investigate the causal relationships between Gut Microbiota and Inflammatory Proteins with RCC, as illustrated in steps 1A and 2A of Fig. 1. Concurrently, we treated RCC as the “exposure” and Gut Microbiota and Inflammatory Proteins as the “outcomes,” selecting SNPs significantly associated with RCC (p < 5 × 10^–6^) as IVs to examine their reverse causal relationships (steps 1B and 2B in Fig. 1).

Based on the analyses from steps 1A and 2A, we included Gut Microbiota and Inflammatory Proteins that exhibited significant causal effects in the subsequent mediation analysis. We specifically analyzed the causal effects of Gut Microbiota on Inflammatory Proteins using MR (step 3, path a, in Fig. 1). Ultimately, through these analytical steps, we determined the mediation proportion of Inflammatory Proteins as mediators.

For our primary analysis, we utilized the inverse variance weighted (IVW) method and conducted the Wald ratios test [24]. The MR results are presented as odds ratios (ORs) accompanied by their corresponding 95% confidence intervals (CIs). Results were deemed statistically significant when IVW p < 0.05 and when the directions of IVW and MR-Egger analyses were consistent. To test for horizontal pleiotropy, we employed MR-PRESSO and MR-Egger regression, while Cochran’s Q test was used to assess heterogeneity for each SNP [25]. We generated MR scatter plots to illustrate SNP-exposure and SNP-outcome associations and performed a leave-one-out analysis to evaluate the influence of each SNP on the results [26]. All analyses were conducted using R (v4.2.3) statistical software along with the "TwoSampleMR" package.

## Results

### Instrumental variable selection

At a significance level of p < 5 × 10^–6^, we identified 5,259 SNPs associated with 471 gut microbiota taxa across five different taxonomic levels: 11 phyla, 24 orders, 62 families, 146 genera, and 209 species. These SNPs were selected as IVs for the 471 gut microbiota taxa (see Additional file 2: Table S1). Similarly, at the same significance level, we identified 1,818 SNPs associated with 91 inflammatory proteins (refer to Additional file 3: Table S2).

### Causal relationship between gut microbiota and RCC

**Fig. 2:**
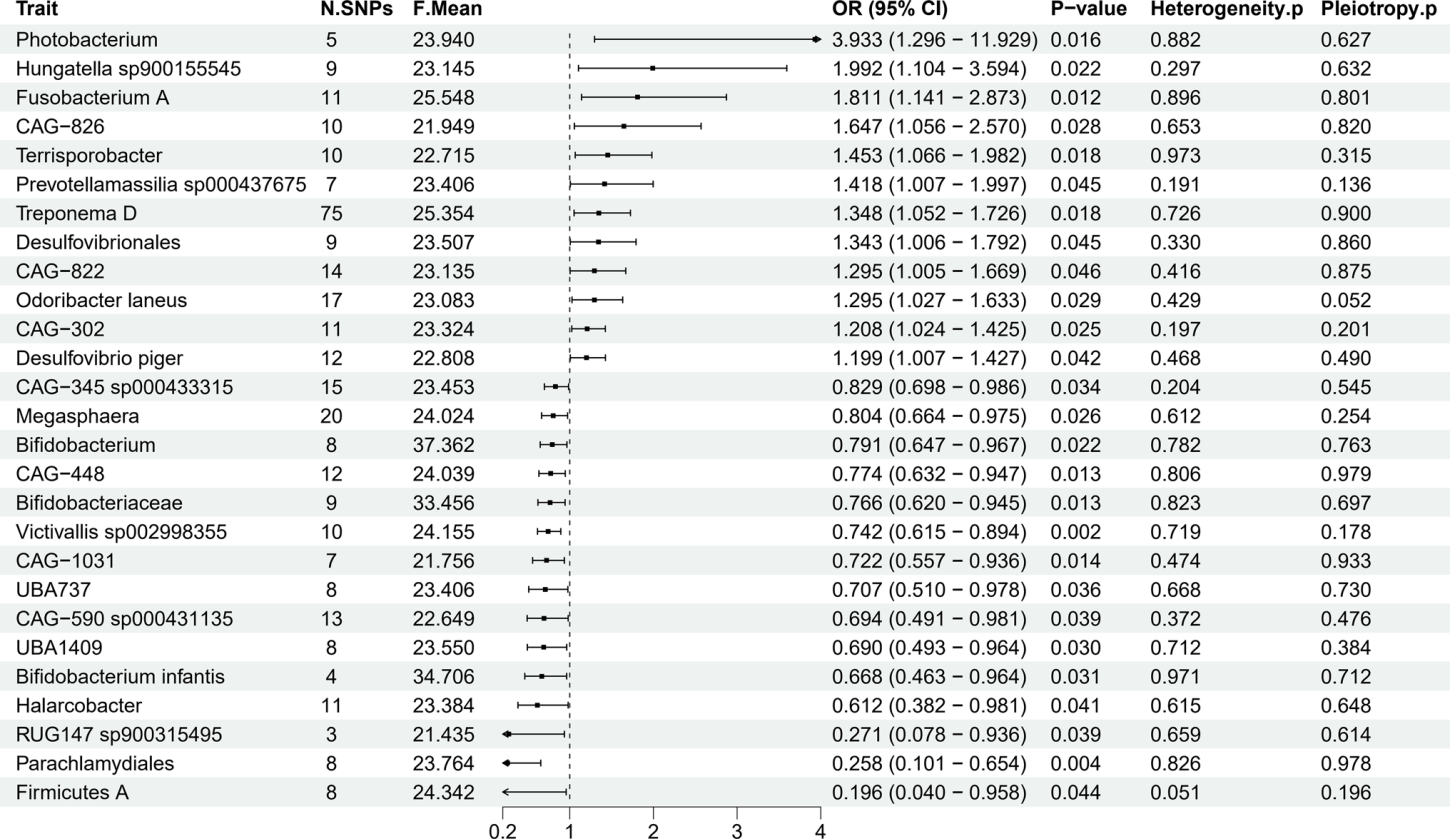
Mendelian randomization results of causal effects between gut microbiotas and RCC

We utilized the IVW method as our primary analysis technique and employed the Wald ratio test. The results are expressed as odds ratios (ORs) accompanied by their corresponding 95% confidence intervals (CIs). Statistical significance is defined by an IVW p-value of less than 0.05, with consistent directional results between IVW and MR-Egger analyses. Our MR results indicate that 27 gut microbiota taxa have a causal relationship with RCC (see Additional file 4: Table S3, Fig. 2). Additional file 5: Table S4 provides detailed information on the 340 SNPs associated with these 27 gut microbiota taxa.

As shown in Fig. 2, MR analysis indicates that the genetic prediction of 12 gut microbiota taxa is associated with an increased risk of RCC. Specifically, the Vibrionaceae Photobacterium group (OR = 3.932, 95% CI 1.296–11.929, p = 0.016), Hungatella sp900155545 from the Clostridiaceae Hungatella group (OR = 1.992, 95% CI 1.104–3.594, p = 0.022), and Fusobacterium A from the Clostridiaceae Firmicutes group (OR = 1.811, 95% CI 1.141–2.873, p = 0.012) are significantly associated with an increased risk of RCC. Conversely, the genetic prediction of 15 gut microbiota taxa is linked to a reduced risk of RCC. Notably, Firmicutes A (OR = 0.196, 95% CI 0.040–0.958, p = 0.044), Parachlamydiales within the Order Chlamydiales (OR = 0.257, 95% CI 0.101–0.654, p = 0.004), and RUG147 sp900315495 in Firmicutes (OR = 0.271, 95% CI 0.078–0.936, p = 0.039) show significant associations with a decreased risk of RCC.

### Causal relationship between inflammatory proteins and RCC

**Fig. 3:**
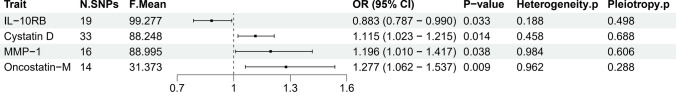
Mendelian randomization results of causal effects between inflammatory proteins and RCC

We employed the IVW method as our primary analysis technique. Results are deemed statistically significant when the IVW p-value is less than 0.05 and when the directions of the IVW and MR-Egger analyses are consistent. The MR results indicate that four inflammatory proteins have a causal relationship with RCC (see Additional file 6: Table S5, Fig. 3). Additional file 7: Table S6 provides detailed information regarding the 84 SNPs associated with these four inflammatory proteins.

As illustrated in Fig. 3, MR analysis suggests that the genetic prediction of three inflammatory proteins is linked to an increased risk of RCC. Specifically, Oncostatin-M (OR = 1.277, 95% CI 1.062–1.537, p = 0.009), MMP-1 (OR = 1.196, 95% CI 1.010–1.417, p = 0.038), and Cystatin D (OR = 1.115, 95% CI 1.023–1.215, p = 0.014) are significantly associated with an elevated risk of RCC. Conversely, IL-10RB (OR = 0.883, 95% CI 0.787–0.990, p = 0.033) is significantly associated with a decreased risk of RCC.

### Causal effects of gut microbiota on inflammatory proteins

We employed the IVW method as our primary analysis technique. Results are deemed statistically significant when the IVW p-value is less than 0.05 and the directions of the IVW and MR-Egger analyses are consistent. Our MR results indicate causal relationships between three pairs of gut microbiota and inflammatory proteins (see Additional file 8: Table S7). Additional file 9: Table S8 provides comprehensive details on the 34 SNPs associated with these three gut microbiota taxa. The MR analysis reveals that CAG-302 is positively associated with Oncostatin-M (OR = 1.115, 95% CI 1.050–1.183, p = 0.0004), and Terrisporobacter is positively associated with MMP-1 (OR = 1.203, 95% CI 1.032–1.403, p = 0.0182). Conversely, CAG-590 sp000431135 shows a negative association with IL-10RB (OR = 0.857, 95% CI 0.747–0.985, p = 0.0294).

### Reverse causal effects of RCC on gut microbiota and inflammatory proteins

As demonstrated in Additional file 10: Table S9, the reverse MR analysis of the positive causal effects of gut microbiota and inflammatory proteins on RCC, derived from the previous MR analysis, indicates that no reverse causal effects exist between these variables.

### Proportion of gut microbiota and RCC association mediated by inflammatory proteins

**Fig. 4:**
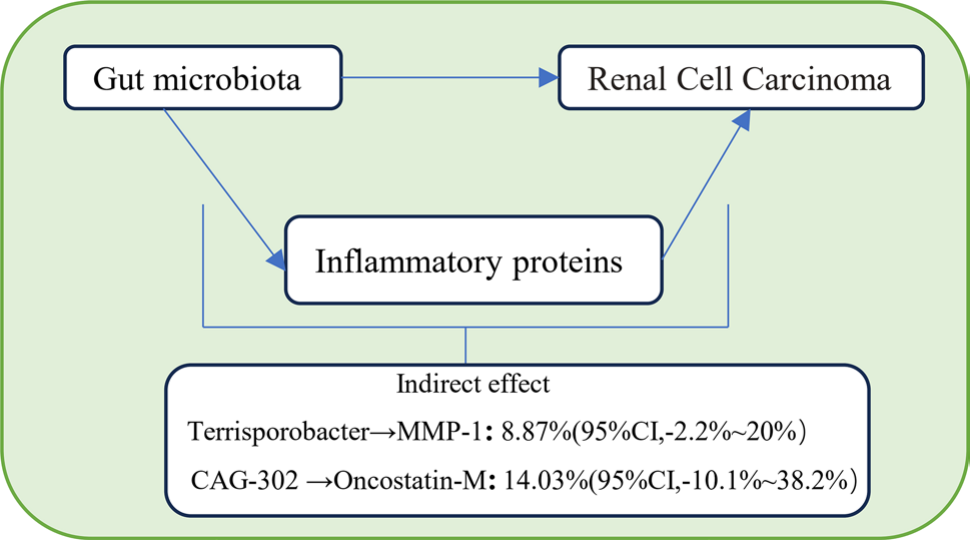
Schematic diagram of the inflammatory proteins mediation effect

We analyzed the role of inflammatory proteins as mediators in the pathway from gut microbiota to RCC (Additional file 11: Table S10). As shown in Fig. 4, we found that Terrisporobacter is associated with increased levels of MMP-1, which, in turn, are linked to an elevated risk of RCC. MMP-1 accounts for 8.87% of the increased risk of RCC associated with Terrisporobacter (mediation proportion: 8.87%; 95% CI − 2.2 to 20%). Similarly, CAG-302 is associated with heightened levels of Oncostatin-M, which is also correlated with an increased risk of RCC. Oncostatin-M accounts for 14.03% of the increased risk of RCC associated with CAG-302 (mediation proportion: 14.03%; 95% CI − 10.1 to 38.2%).

### Sensitivity analyses

According to the MR-Egger regression intercept method, genetic pleiotropy does not bias the results. The MR-PRESSO analysis confirms the absence of horizontal pleiotropy in the MR study (p > 0.05), and Cochran's Q test indicates no significant heterogeneity (p > 0.05) (see Additional files 4, 6, 8, and 10). The leave-one-out analysis further supports the reliability of the MR analysis, as the empty lines do not fall within the total confidence interval of the SNPs (refer to Additional file 1: Figure S1). The scatter plot illustrates the overall impact of Gut Microbiota on RCC (see Additional file 1: Figure S2). Additionally, the forest plot demonstrates the causal relationship between Gut Microbiota and RCC (refer to Additional file 1: Figure S3).

## Discussion

The gut microbiome influences renal physiology through various mechanisms, including the induction of chronic inflammation, the production of carcinogenic metabolites, and the disruption of the gut-kidney axis. Gut microbiota can generate inflammatory mediators, such as endotoxins and pro-inflammatory bacterial metabolites, which are transported to the kidneys via the bloodstream, thereby inducing chronic inflammation [27, 28]. For instance, lipopolysaccharides (LPS) produced by gut microbiota can activate the Toll-like receptor 4 (TLR4) pathway of the immune system, leading to an increased release of pro-inflammatory cytokines such as tumor necrosis factor (TNF) and interleukin-6 (IL-6). This cascade promotes an inflammatory environment that is conducive to the survival and proliferation of renal cancer cells [29]. Additionally, certain gut bacteria can metabolize meat to produce harmful compounds, such as nitrosamines, which enter the kidneys through the bloodstream and interact with renal cell DNA, resulting in genetic mutations that elevate the risk of renal cancer [30]. The complex biological connection between the gut and the kidneys, known as the gut-kidney axis, is significant; when the gut barrier is compromised, bacteria and toxins can more readily enter the bloodstream and reach the kidneys, potentially promoting renal pathology and triggering immune system attacks on renal cells, thereby increasing the risk of renal cancer [31]. However, due to the complexity of renal cancer and the intricacies of the gut microbiome, establishing clear relationships through observational studies alone remains a challenge.

In this study, we employed MR analysis to explore the causal relationships between 471 gut microbiota taxa and 91 inflammatory proteins in relation to RCC. Our findings indicate that certain gut microbiota taxa and inflammatory proteins serve as risk factors, while others act as protective factors. Furthermore, we identified two axes of gut microbiota that facilitate the development of RCC through inflammatory proteins.

The presence of Photobacterium in the gut microbiota and its direct association with human diseases, including tumors, has not been extensively investigated. Hungatella sp900155545, an anaerobic bacterium within the gut microbiota, exhibits increased abundance in the gut microbiota of breast cancer patients [32]. Bacteria from the genus Fusobacterium, particularly Fusobacterium nucleatum (F. nucleatum), have been found in high quantities in the tumor tissues of colorectal cancer patients. These bacteria undermine the anti-tumor adaptive immune response by inhibiting T cell proliferation and inducing T cell apoptosis, potentially accelerating colorectal cancer progression [33]. Our study demonstrates that these three gut microbiota taxa exhibit the strongest positive correlation with RCC, while the following three taxa show the most significant negative correlation. Short-chain fatty acids (SCFAs) produced by Firmicutes, such as butyrate, possess anti-inflammatory properties. A reduction in Firmicutes abundance may compromise gut barrier function and contribute to chronic inflammation. This decrease in Firmicutes is significantly associated with the onset of chronic liver disease and hepatocellular carcinoma [34]. Currently, there is limited research on bacteria from the genus Parachlamydiales and the specific strain RUG147 sp900315495 within the gut microbiota. The mechanisms by which these bacteria influence RCC warrant further investigation.

This study demonstrates that the inflammatory factor MMP-1 mediates the pro-carcinogenic effects of the gut microbiota Terrisporobacter on RCC. MMP-1, or matrix metalloproteinase-1, is capable of degrading the extracellular matrix (ECM) and plays a significant role in both inflammation and tumor progression. Overexpression of MMP-1 is linked to poor prognoses in lung cancer, breast cancer, and various other malignancies [35]. The abundance of Terrisporobacter, a member of the class Clostridia, is elevated in thyroid cancer patients and is thought to increase cancer risk through mechanisms involving inflammation and immune regulation [36]. Mark R. Schmitt et al. found that an imbalance of Terrisporobacter may promote colorectal cancer via immune regulation and inflammatory pathways [37]. Current research indicates that tumor-associated macrophages (TAMs) secrete various inflammatory cytokines, such as IL-1β, which facilitate the invasion of RCC by activating MMP-1 and other matrix metalloproteinases [38]. Jiang et al.'s study on MMP-1 inhibitors suggests that reducing MMP-1 activity may slow the progression of RCC [39]. However, direct research on the relationship between Terrisporobacter and RCC remains limited.

This study demonstrates that the inflammatory factor Oncostatin-M mediates the pro-carcinogenic effects of the gut microbiota strain CAG-302 on RCC. Oncostatin M (OSM) is a pro-inflammatory cytokine belonging to the IL-6 family. Masjedi et al. have shown that in various tumors, including breast, liver, and gastric cancers, OSM can activate the JAK/STAT signaling pathway—particularly STAT3—thereby enhancing cancer cell invasiveness, metastasis, and resistance to treatment, which promotes tumor invasion and metastasis [40]. Nguyen-Tran et al. have also found that OSM facilitates the progression and metastasis of clear cell Renal Cell Carcinoma (ccRCC) within an inflammatory microenvironment [41]. Research on the bacterium CAG-302 and its associations with human diseases, tumors, and RCC remains limited and warrants further investigation.

This study represents the first large-scale MR analysis exploring the causal relationships between gut microbiota, inflammatory proteins, and RCC, utilizing the most recent large-sample GWAS data. However, our study has certain limitations, including the fact that the sample source for RCC is drawn from the Finnish population.

## Conclusion

In this study, we comprehensively explored the causal relationships among gut microbiota, inflammatory proteins, and RCC. We identified 12 positive and 15 negative causal relationships between the genetic liability of gut microbiota and RCC. Additionally, there are three positive and one negative causal relationships between inflammatory proteins and RCC. Importantly, we found no bidirectional causal relationships among gut microbiota, inflammatory proteins, and RCC. Furthermore, we identified two axes of relationships in which gut microbiota promote the development of RCC through inflammatory proteins acting as mediators.

## Supplementary Information

Additional file 1 (DOCX 6187 KB) The plots of MR analysis results.

Additional file 2 (XLSX 503 KB) 5259 SNPs for the 471 gut microbiota taxa.

Additional file 3 (XLSX 228 KB) 1818 SNPs for the 91 Inflammatory Proteins.

Additional file 4 (XLSX 14 KB) The causal effects of gut microbiota on Renal Cell Carcinoma.

Additional file 5 (XLSX 68 KB) The characteristics of 340 SNPs analyzing the causal effects of the gut microbiota on Renal Cell Carcinoma.

Additional file 6 (XLSX 11 KB) The causal effects of Inflammatory Proteins on Renal Cell Carcinoma.

Additional file 7 (XLSX 24 KB) The characteristics of 84 SNPs analyzing the causal effects of the Inflammatory Proteins on Renal Cell Carcinoma.

Additional file 8 (XLSX 11 KB) The causal effects of Gut Microbiota on Inflammatory Proteins.

Additional file 9 (XLSX 16 KB) The characteristics of 34 SNPs analyzing the causal effects of the Gut Microbiota on Inflammatory Proteins.

Additional file 10 (XLSX 15 KB) The causal effects of Renal Cell Carcinoma on Gut Microbiota and Inflammatory Proteins.

Additional file 11 (XLSX 19 KB) Causal effects of gut microbiota associated with Renal Cell Carcinoma on Inflammatory Proteins associated with Renal Cell Carcinoma.

## Data Availability

All data used in this study are included in the article and its supplementary materials. For additional data requests, please contact the corresponding author. The statistical code required to reproduce the results is also provided within the article or is publicly accessible as indicated.

## Abbreviations

RCC: Renal cell carcinoma
MR: Mendelian randomization
GWAS: Genome-wide association study
IVs: Instrumental variables
CI: Confidence interval
IVW: Inverse variance weighting
MR-PRESSO: MR pleiotropy residual sum and outlier
OR: Odds ratio
SNPs: Single nucleotide polymorphisms
MMP-1: Matrix metalloproteinase-1
OSM: Oncostatin M
TLR4: Toll-like receptor 4
TNF: Tumor necrosis factor
IL-6: Interleukin-6
LPS: Lipopolysaccharides
