# Supplementary material for: Genetically predicted inflammatory proteins mediate the association between gut microbiota and renal cell carcinoma
Source: Discov Oncol. 2025 Feb 20;16:216. doi: 10.1007/s12672-025-01980-y (PMC11842667; doi:10.1007/s12672-025-01980-y)
Supplement: Supplementary file 1 — Additional file 1 (DOCX 6187 KB) The plots of MR analysis results. [file 12672_2025_1980_MOESM1_ESM.docx]

**Additional Figures 1**

**Figure S1.** MR leave-one-out sensitivity analysis for Gut microbiota on Renal Cell Carcinoma.

**Figure S2.** Scatter plots for the effect of Gut microbiota on Renal Cell Carcinoma.

**Figure S3.** Forest plots for the effect of Gut microbiota on Renal Cell Carcinoma.

**Figure S1**. MR leave-one-out sensitivity analysis for Gut microbiota on Renal Cell Carcinoma.


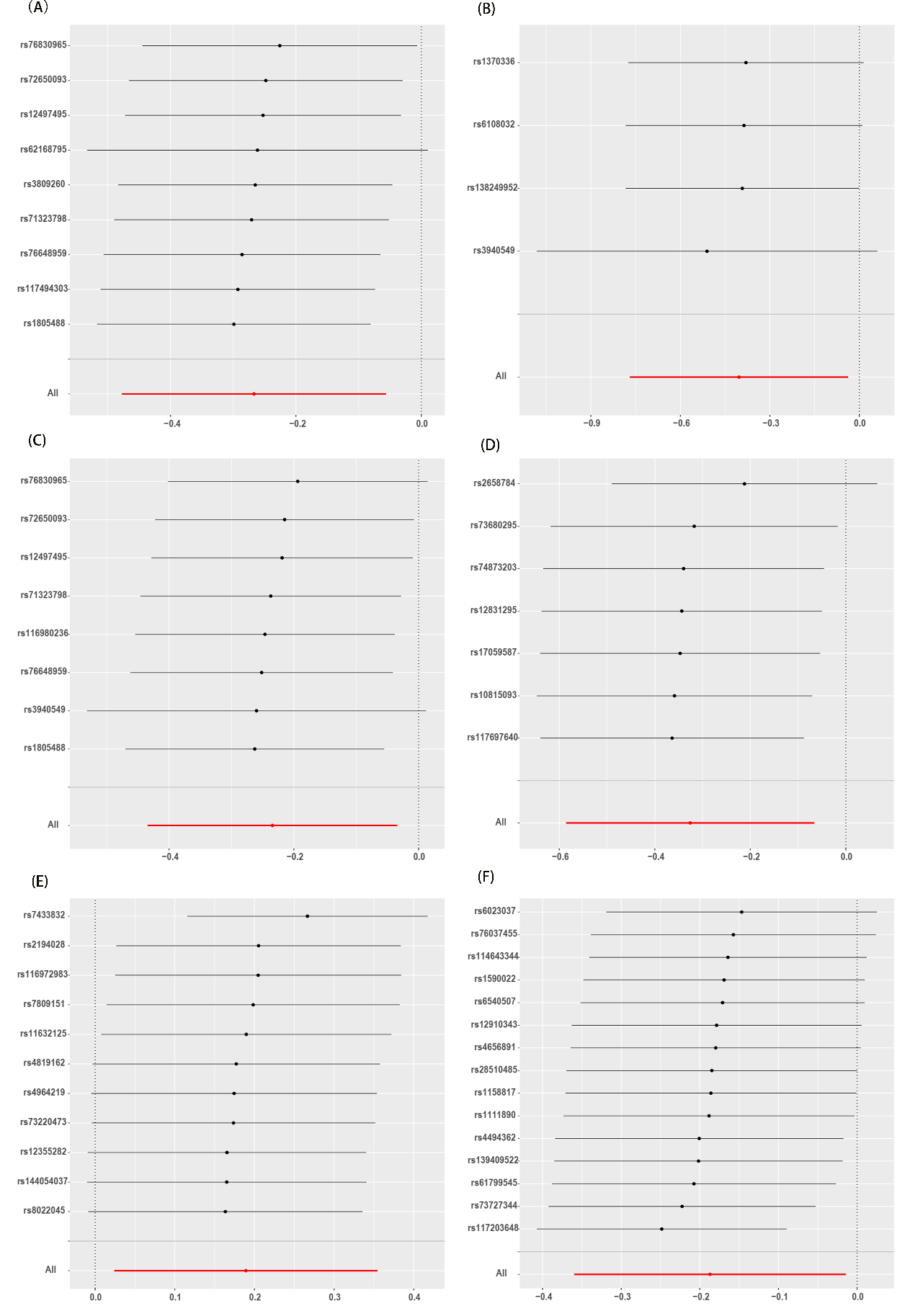


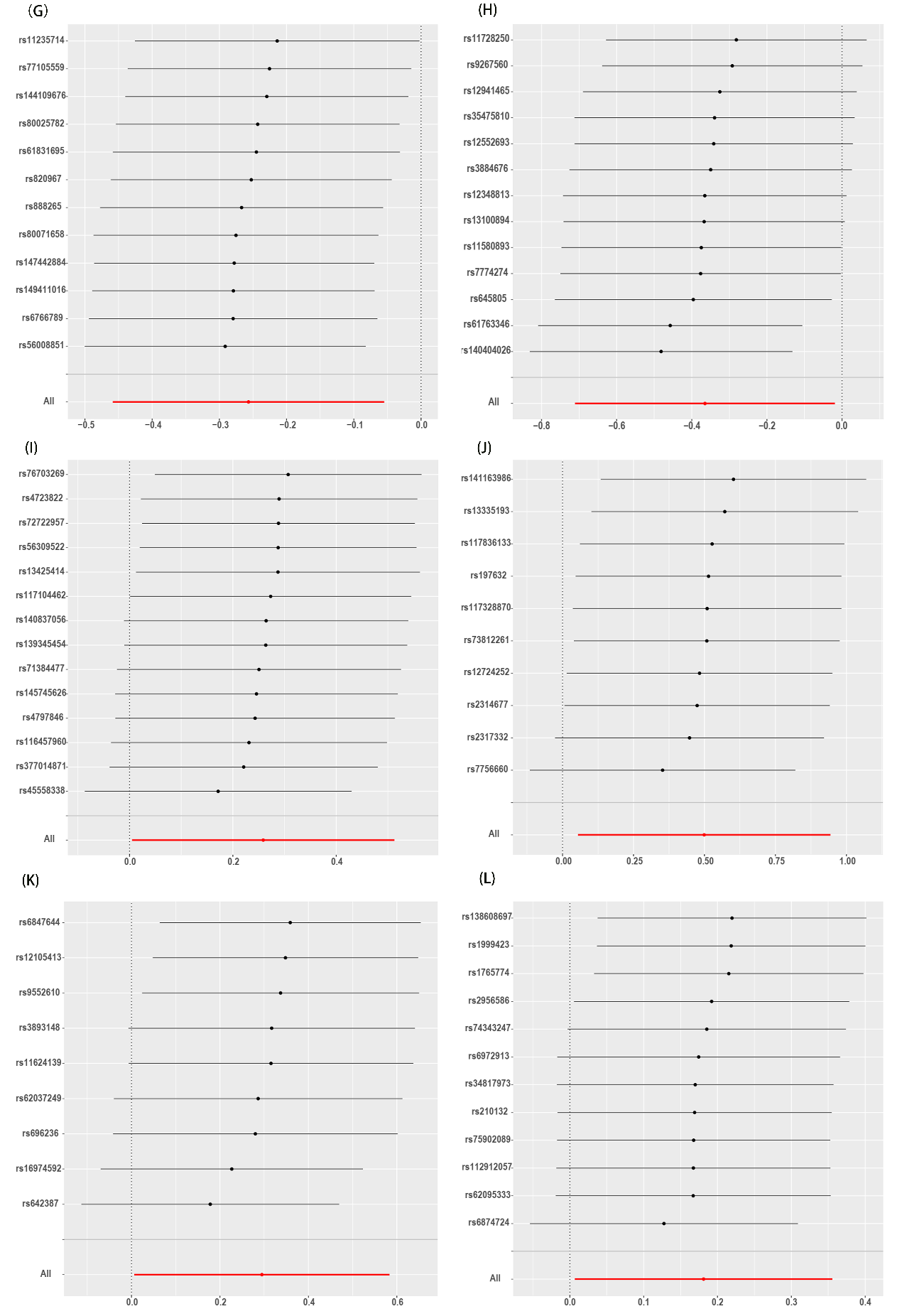


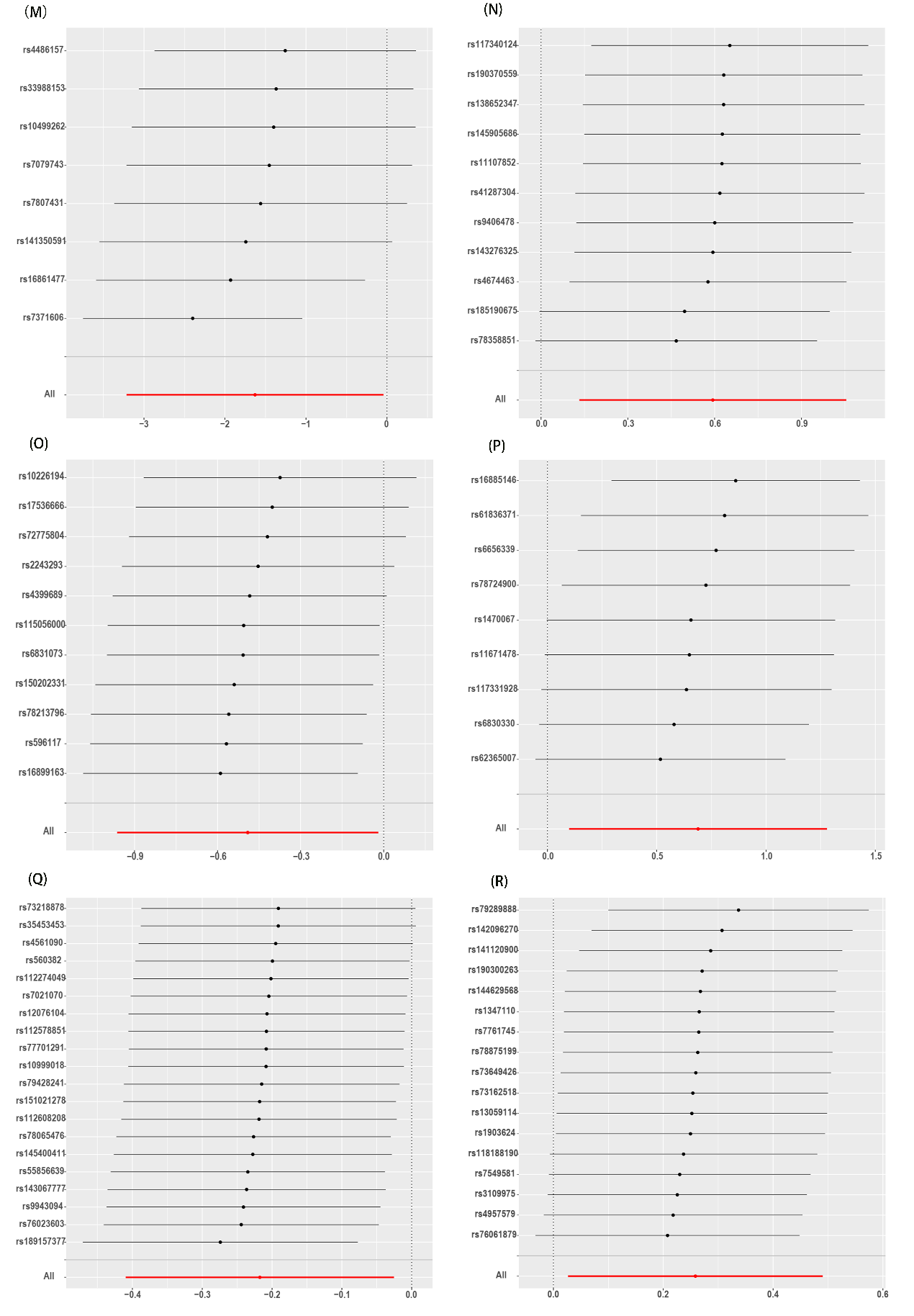


1. Analysis for " Bifidobacteriaceae " on " Renal Cell Carcinoma ".
2. Analysis for " Bifidobacterium infantis " on " Renal Cell Carcinoma ".
3. Analysis for " Bifidobacterium " on " Renal Cell Carcinoma ".
4. Analysis for " CAG-1031 " on " Renal Cell Carcinoma ".
5. Analysis for " CAG-302 " on " Renal Cell Carcinoma ".
6. Analysis for " CAG-345 sp000433315 " on " Renal Cell Carcinoma ".
7. Analysis for " CAG-448 " on " Renal Cell Carcinoma ".
8. Analysis for " CAG-590 sp000431135 " on " Renal Cell Carcinoma ".
9. Analysis for " CAG-822 " on " Renal Cell Carcinoma ".
10. Analysis for " CAG-826 " on " Renal Cell Carcinoma ".
11. Analysis for " Desulfovibrionales " on " Renal Cell Carcinoma ".
12. Analysis for " Desulfovibrio piger " on " Renal Cell Carcinoma ".
13. Analysis for " Firmicutes A " on " Renal Cell Carcinoma ".
14. Analysis for " Fusobacterium A " on " Renal Cell Carcinoma ".
15. Analysis for " Halarcobacter " on " Renal Cell Carcinoma ".
16. Analysis for " Hungatella sp900155545 " on " Renal Cell Carcinoma ".
17. Analysis for " Megasphaera " on " Renal Cell Carcinoma ".
18. Analysis for " Odoribacter laneus " on " Renal Cell Carcinoma ".


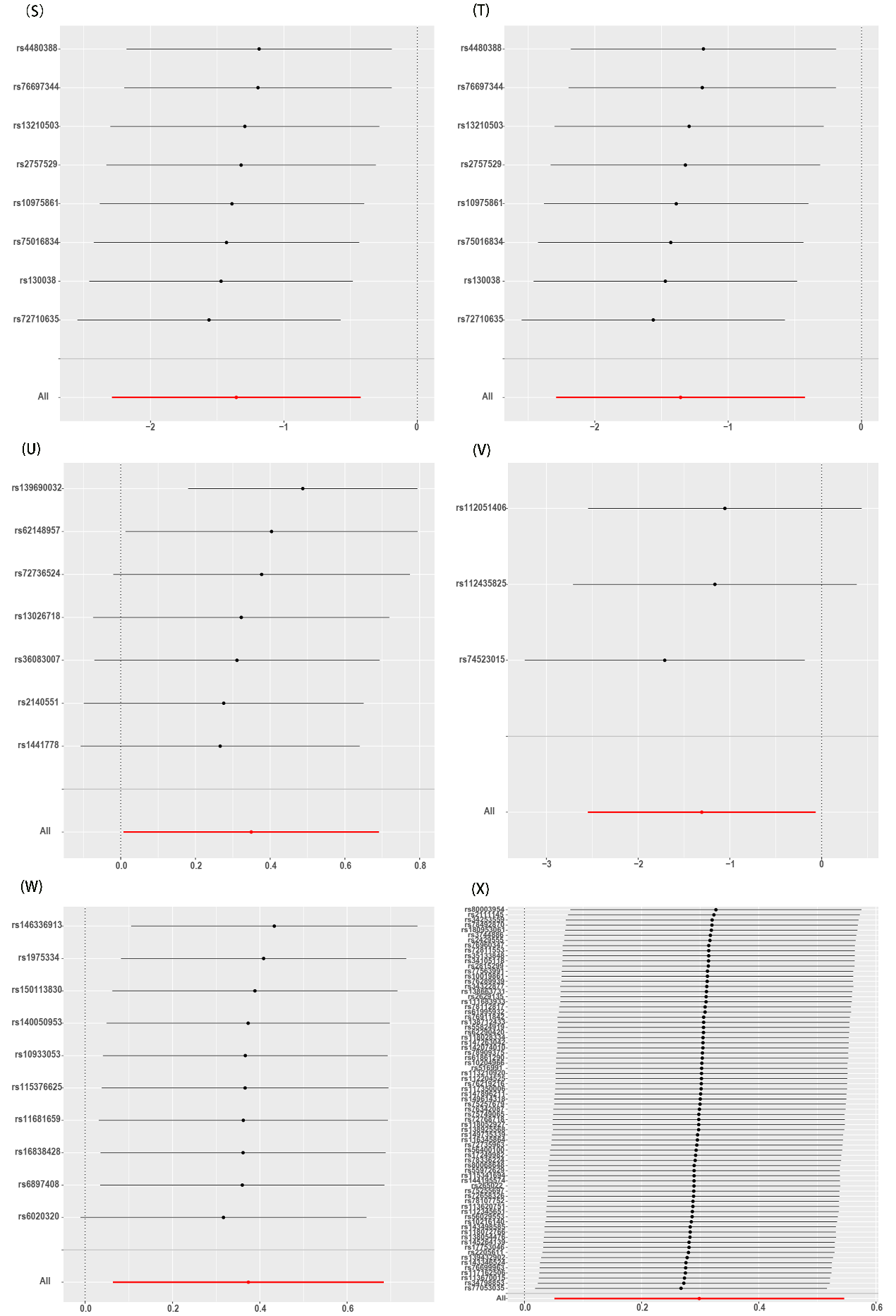


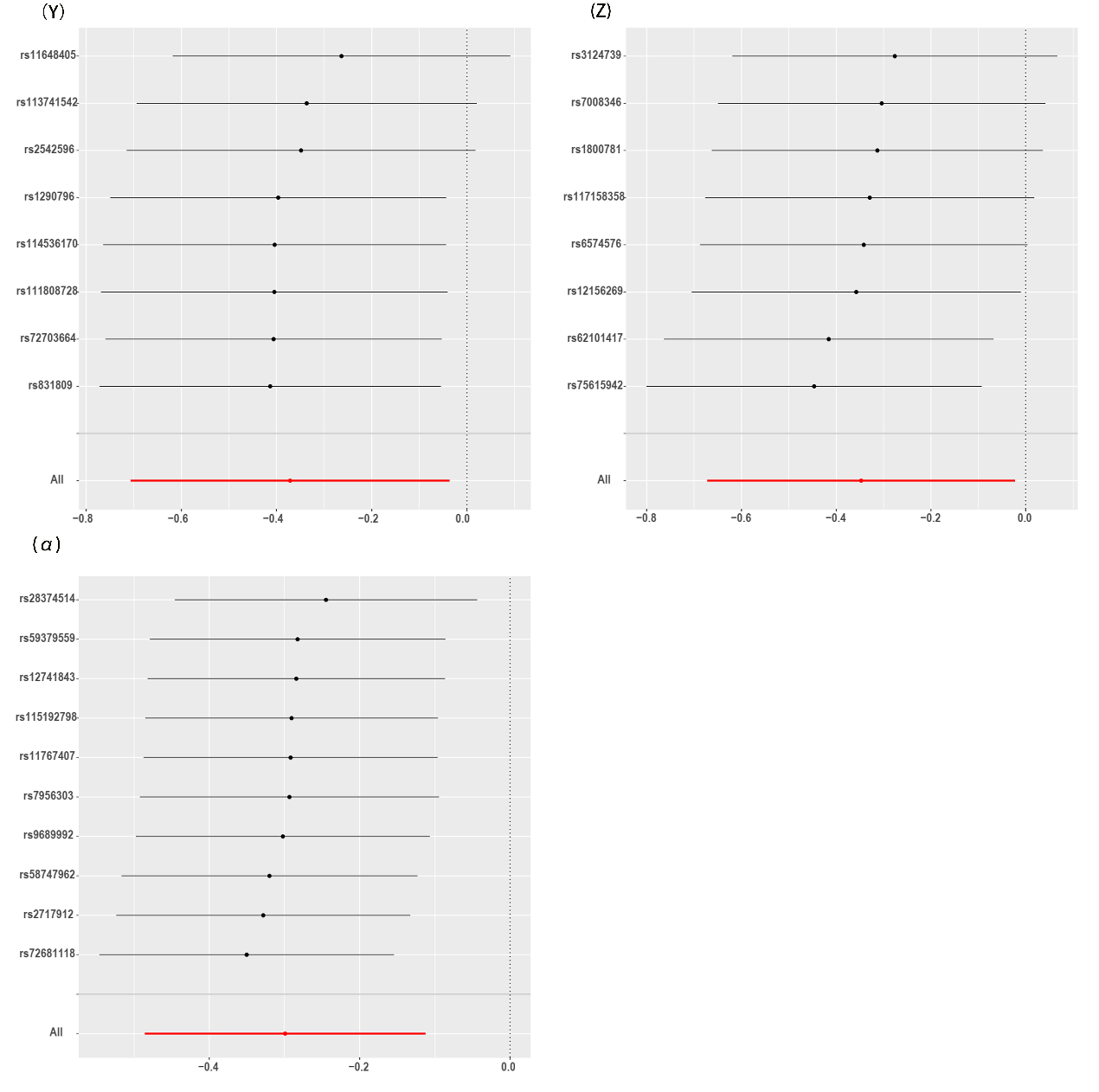


1. Analysis for " Parachlamydiales " on " Renal Cell Carcinoma ".
2. Analysis for " Photobacterium " on " Renal Cell Carcinoma ".
3. Analysis for " Prevotellamassilia sp000437675 " on " Renal Cell Carcinoma ".
4. Analysis for " RUG147 sp900315495 " on " Renal Cell Carcinoma ".
5. Analysis for " Terrisporobacter " on " Renal Cell Carcinoma ".
6. Analysis for " Treponema D " on " Renal Cell Carcinoma ".
7. Analysis for " UBA1409 " on " Renal Cell Carcinoma ".
8. Analysis for " UBA737 " on " Renal Cell Carcinoma ".

(α)Analysis for " Victivallis sp002998355 " on " Renal Cell Carcinoma ".

**Figure S2**. Scatter plots for the effect of Gut microbiota on Renal Cell Carcinoma.


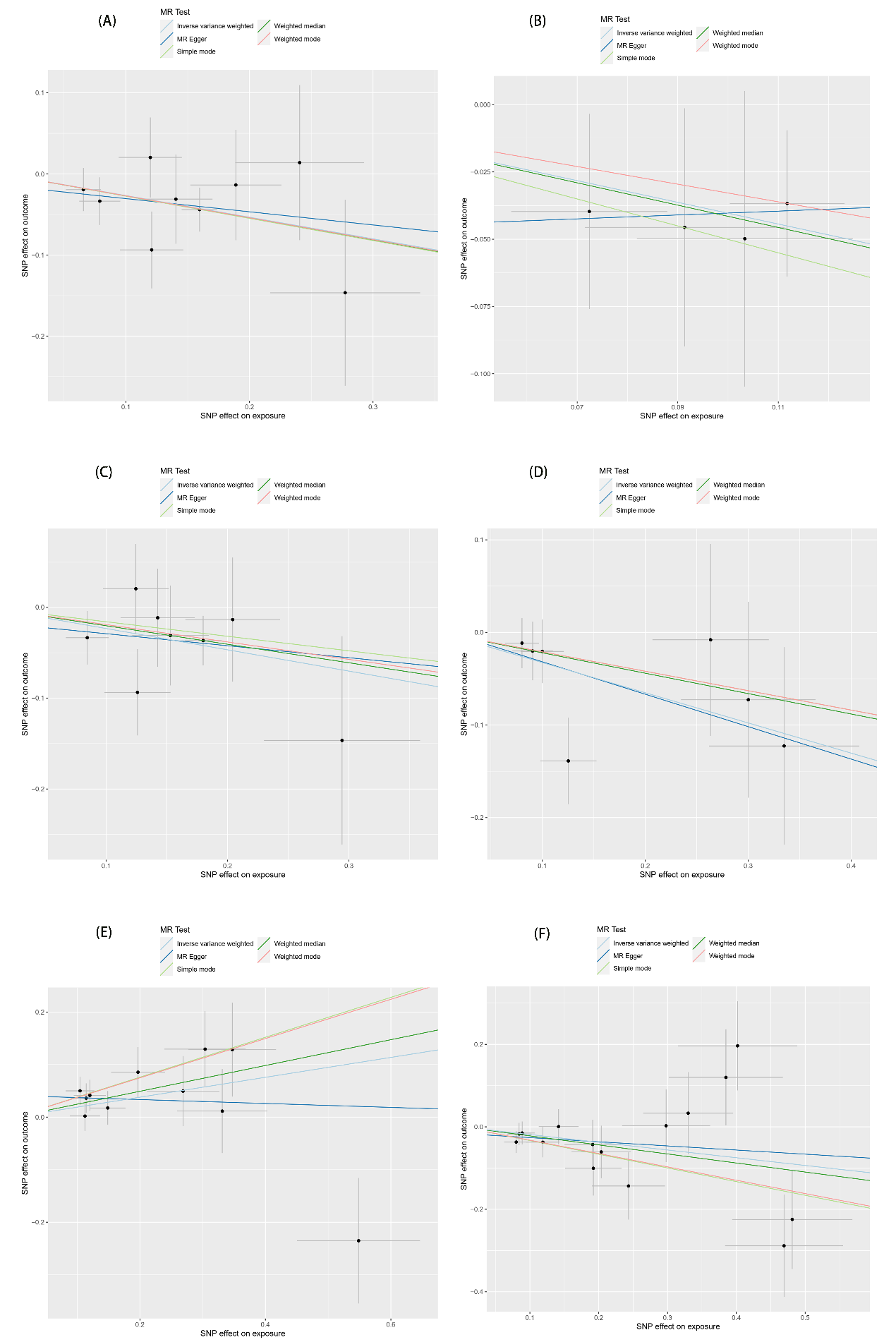


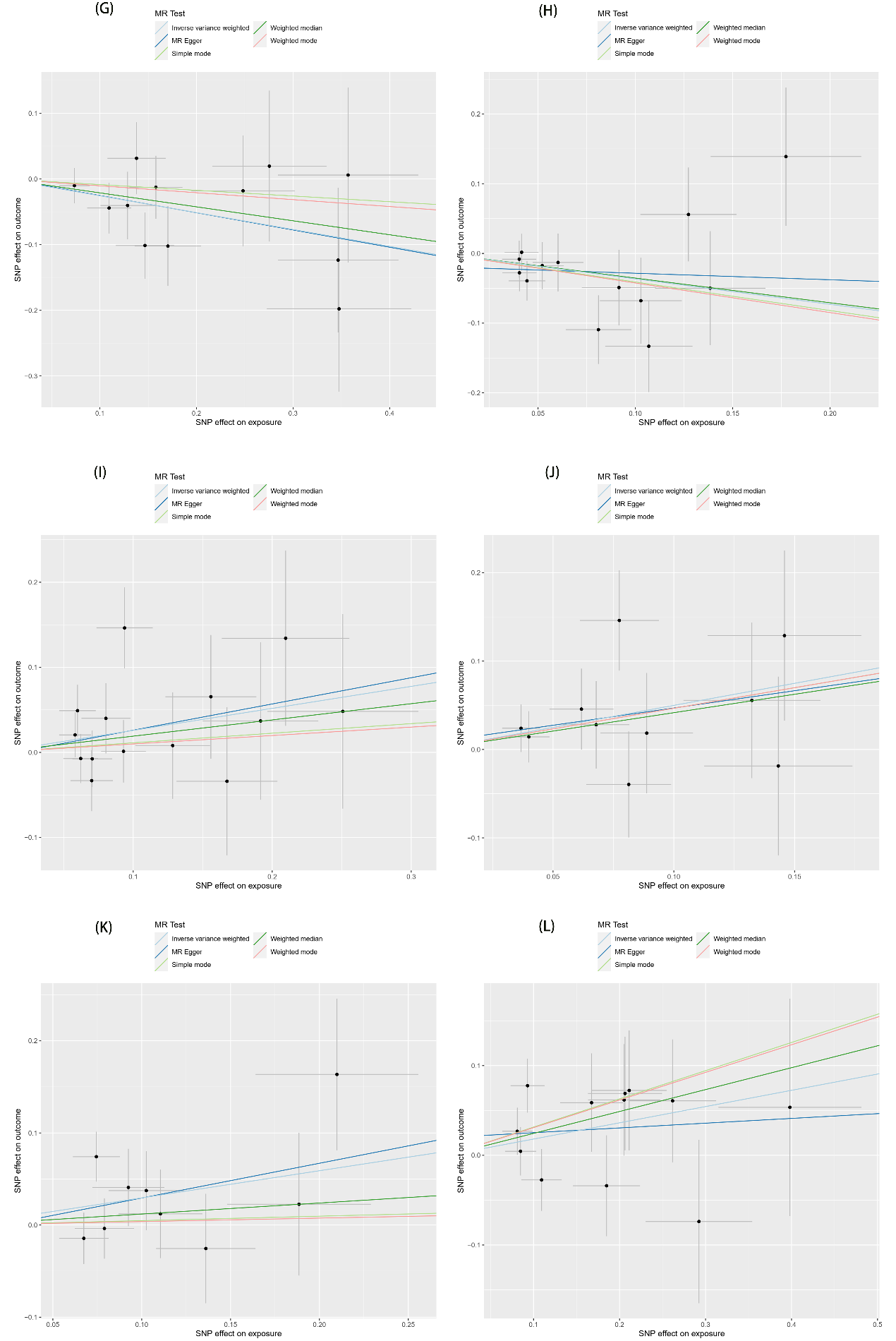


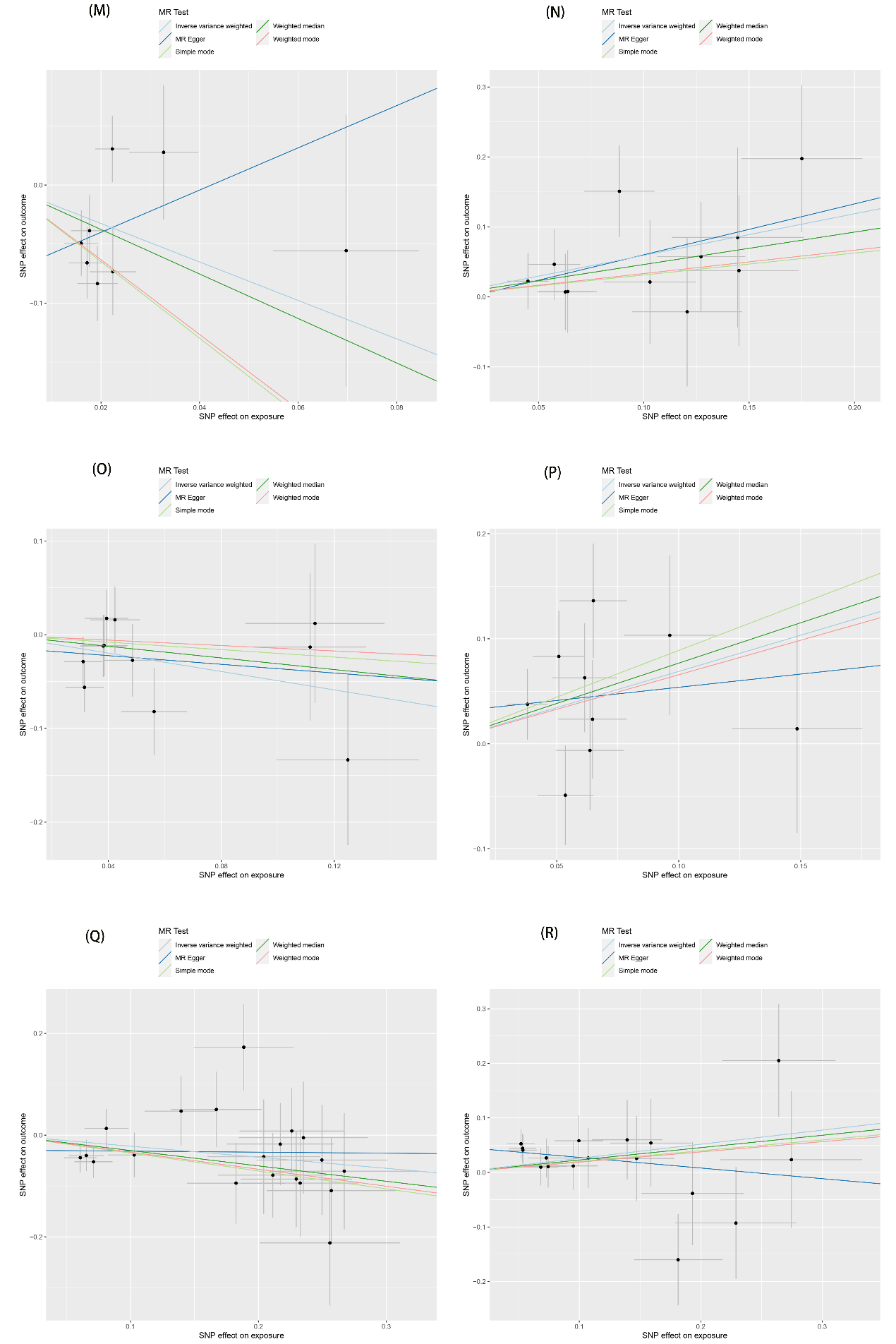


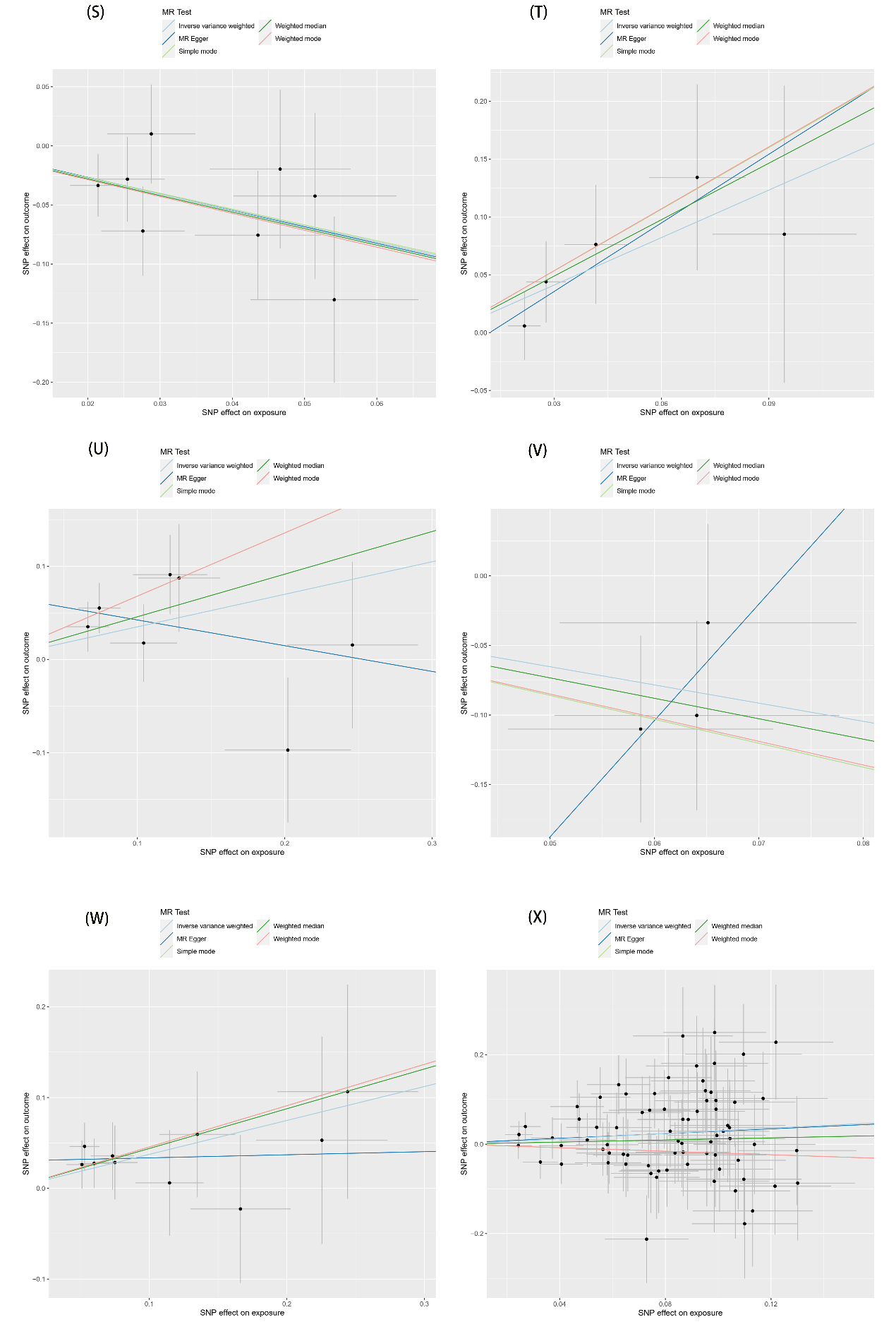


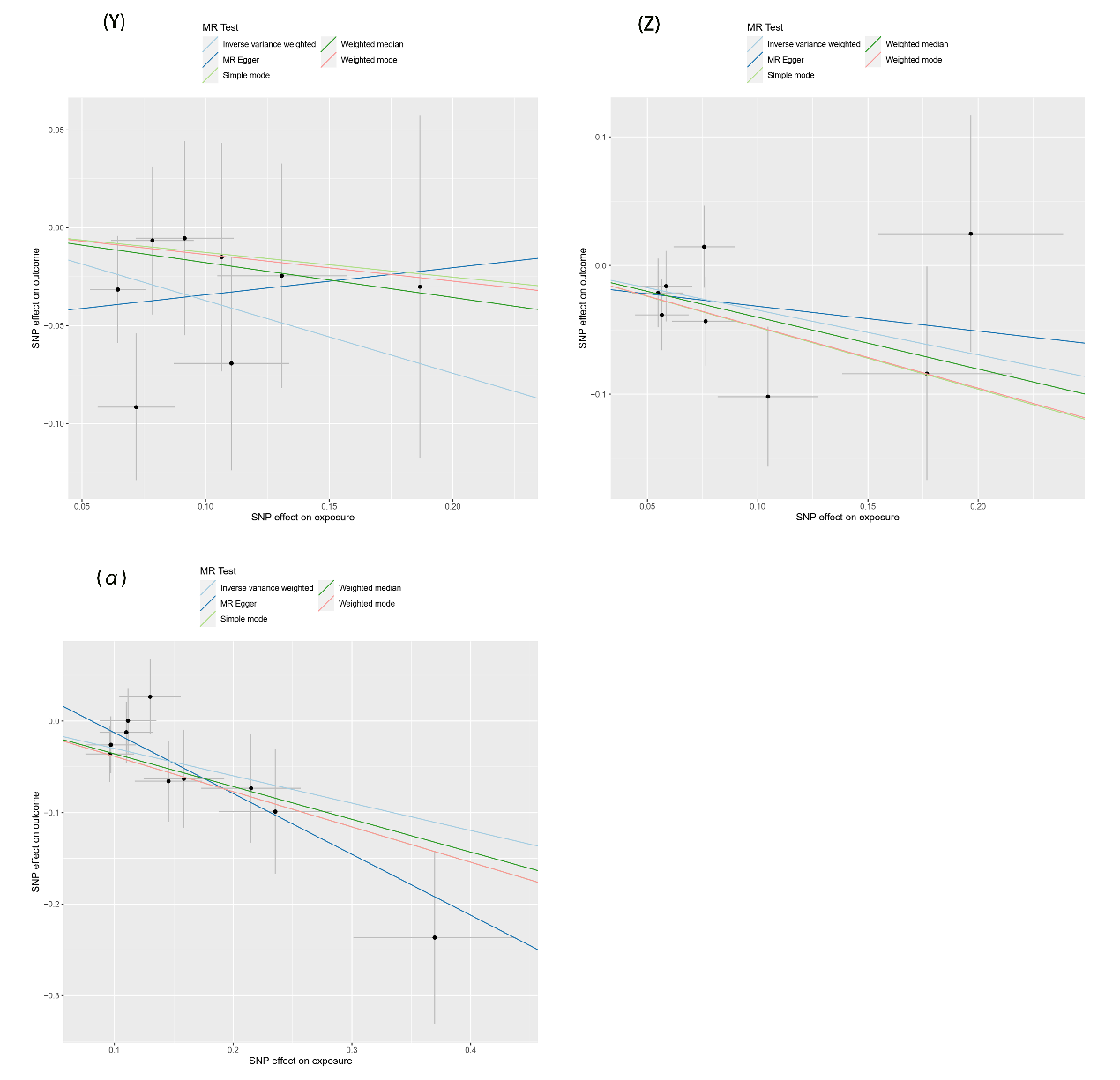


1. Scatter plots for the effect of " Bifidobacteriaceae " on " Renal Cell Carcinoma ".
2. Scatter plots for the effect of " Bifidobacterium infantis " on " Renal Cell Carcinoma ".
3. Scatter plots for the effect of " Bifidobacterium " on " Renal Cell Carcinoma ".
4. Scatter plots for the effect of " CAG-1031 " on " Renal Cell Carcinoma ".
5. Scatter plots for the effect of " CAG-302 " on " Renal Cell Carcinoma ".
6. Scatter plots for the effect of " CAG-345 sp000433315 " on " Renal Cell Carcinoma ".
7. Scatter plots for the effect of " CAG-448 " on " Renal Cell Carcinoma ".
8. Scatter plots for the effect of " CAG-590 sp000431135 " on " Renal Cell Carcinoma ".
9. Scatter plots for the effect of " CAG-822 " on " Renal Cell Carcinoma ".
10. Scatter plots for the effect of " CAG-826 " on " Renal Cell Carcinoma ".
11. Scatter plots for the effect of " Desulfovibrionales " on " Renal Cell Carcinoma ".
12. Scatter plots for the effect of " Desulfovibrio piger " on " Renal Cell Carcinoma ".
13. Scatter plots for the effect of " Firmicutes A " on " Renal Cell Carcinoma ".
14. Scatter plots for the effect of " Fusobacterium A " on " Renal Cell Carcinoma ".
15. Scatter plots for the effect of " Halarcobacter " on " Renal Cell Carcinoma ".
16. Scatter plots for the effect of " Hungatella sp900155545 " on " Renal Cell Carcinoma ".
17. Scatter plots for the effect of " Megasphaera " on " Renal Cell Carcinoma ".
18. Scatter plots for the effect of " Odoribacter laneus " on " Renal Cell Carcinoma ".
19. Scatter plots for the effect of " Parachlamydiales " on " Renal Cell Carcinoma ".
20. Scatter plots for the effect of " Photobacterium " on " Renal Cell Carcinoma ".
21. Scatter plots for the effect of " Prevotellamassilia sp000437675 " on "Renal Cell Carcinoma".
22. Scatter plots for the effect of " RUG147 sp900315495 " on " Renal Cell Carcinoma ".
23. Scatter plots for the effect of " Terrisporobacter " on " Renal Cell Carcinoma ".
24. Scatter plots for the effect of " Treponema D " on " Renal Cell Carcinoma ".
25. Scatter plots for the effect of " UBA1409 " on " Renal Cell Carcinoma ".
26. Scatter plots for the effect of " UBA737 " on " Renal Cell Carcinoma ".

(α) Scatter plots for the effect of " Victivallis sp002998355 " on " Renal Cell Carcinoma ".

**Figure S3. Forest plots for the effect of Gut microbiota on Renal Cell Carcinoma.**


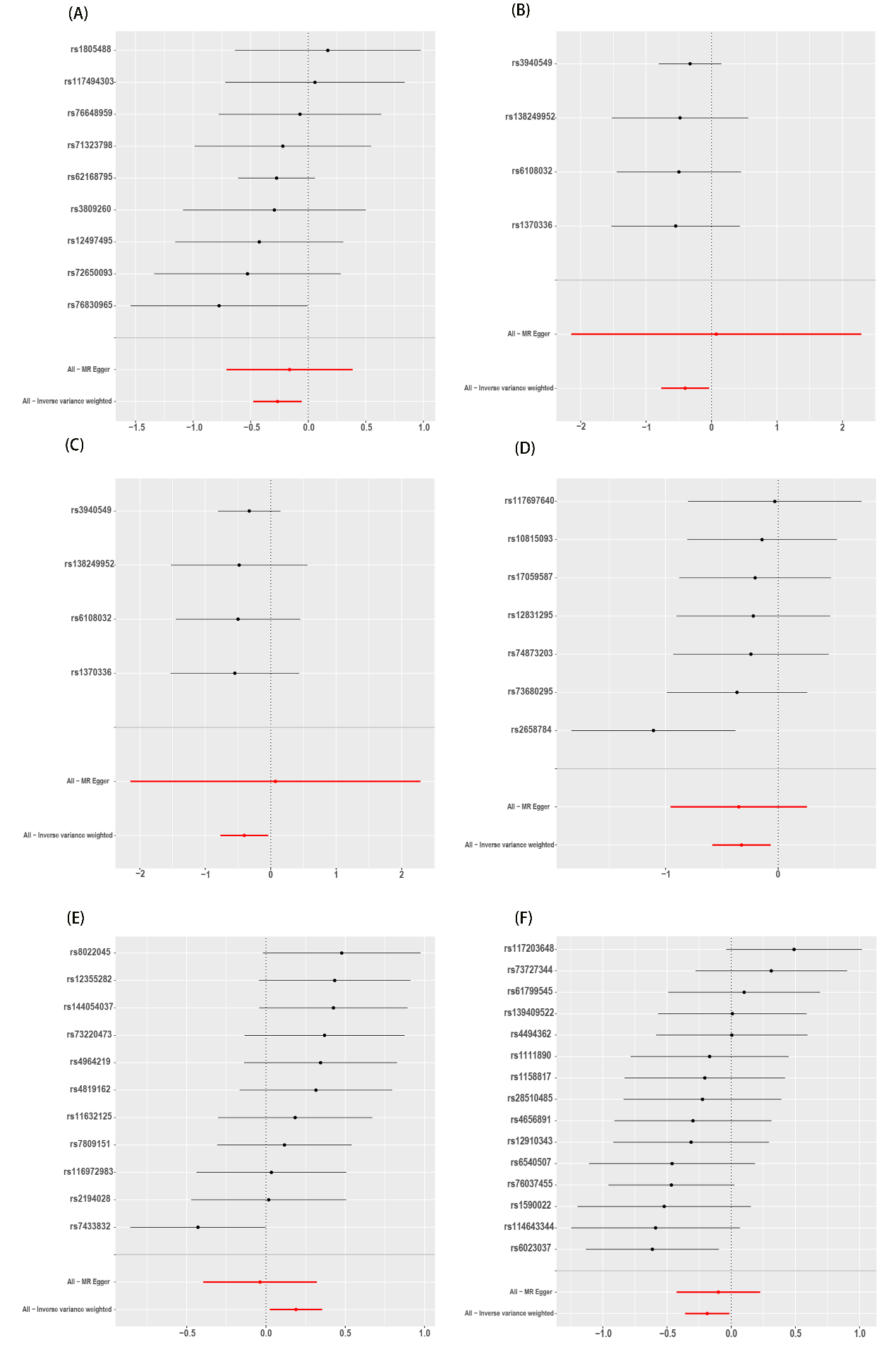


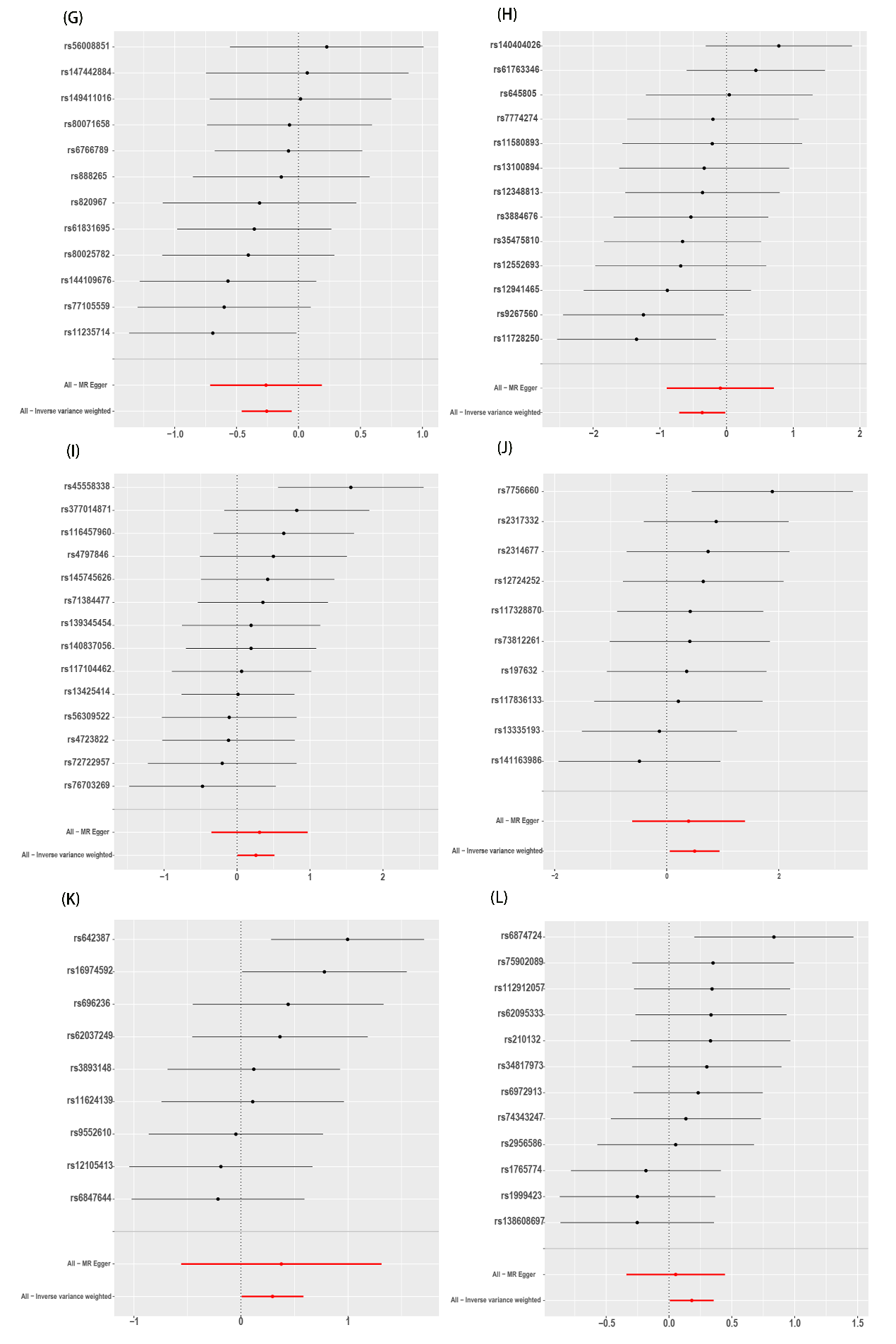


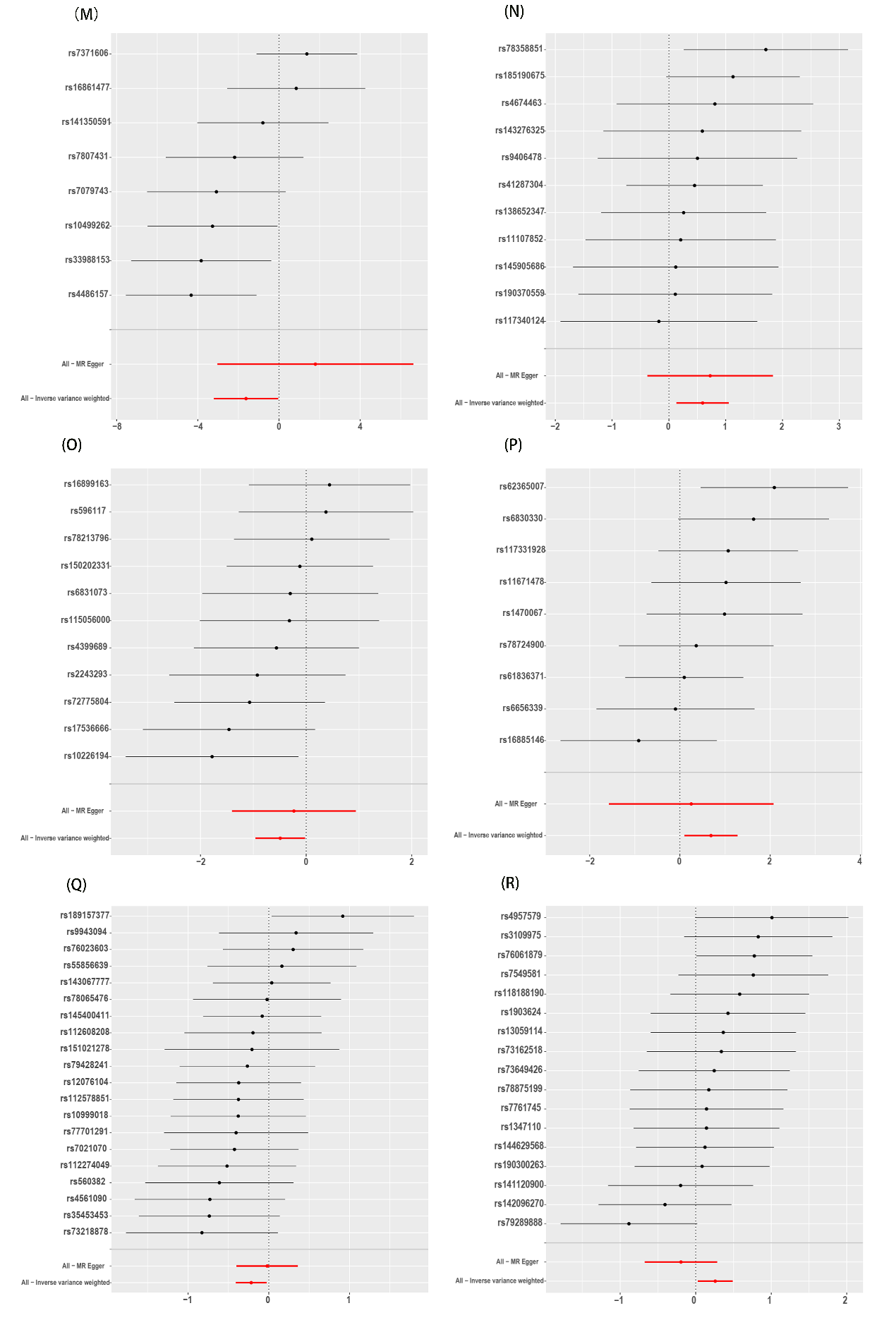


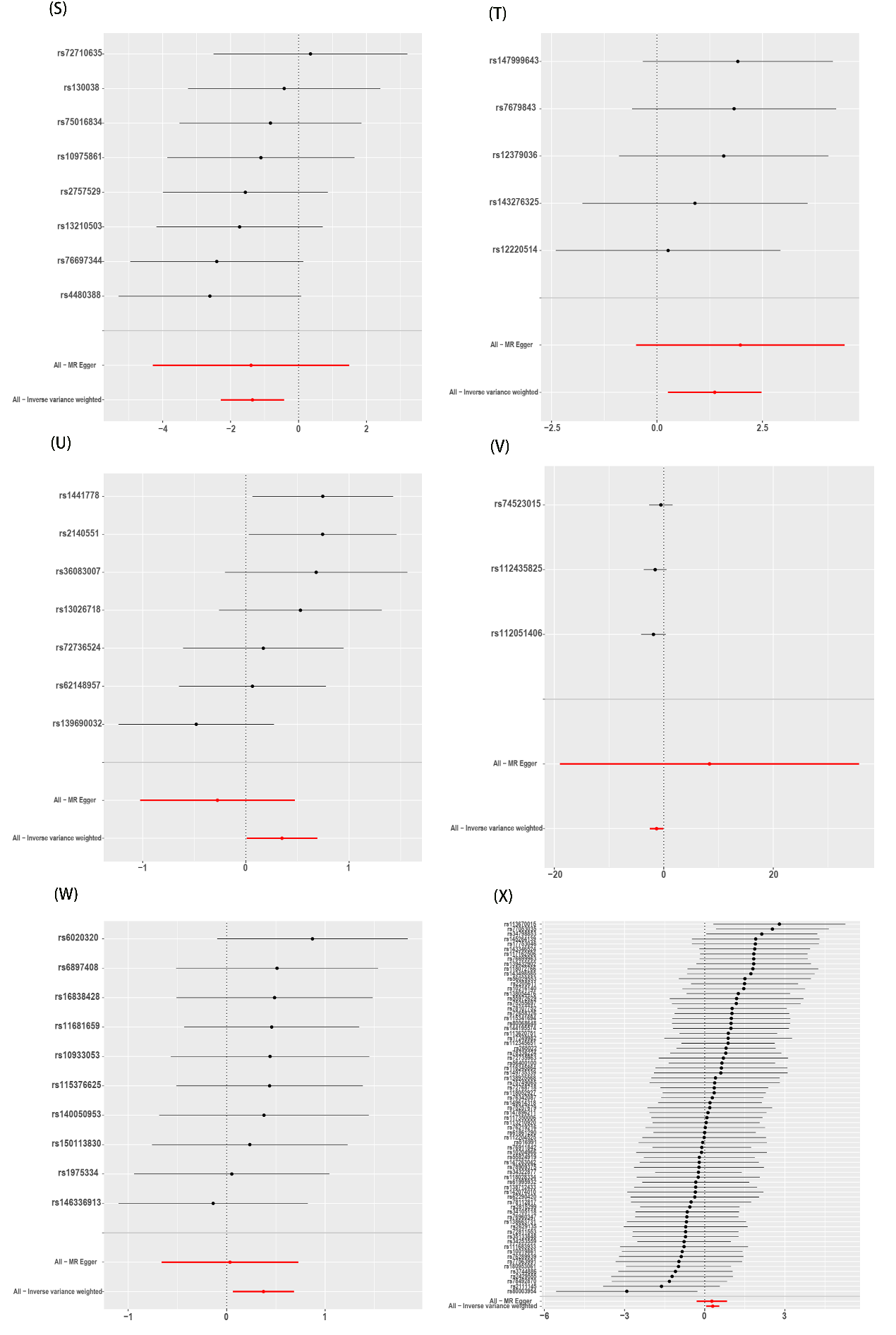


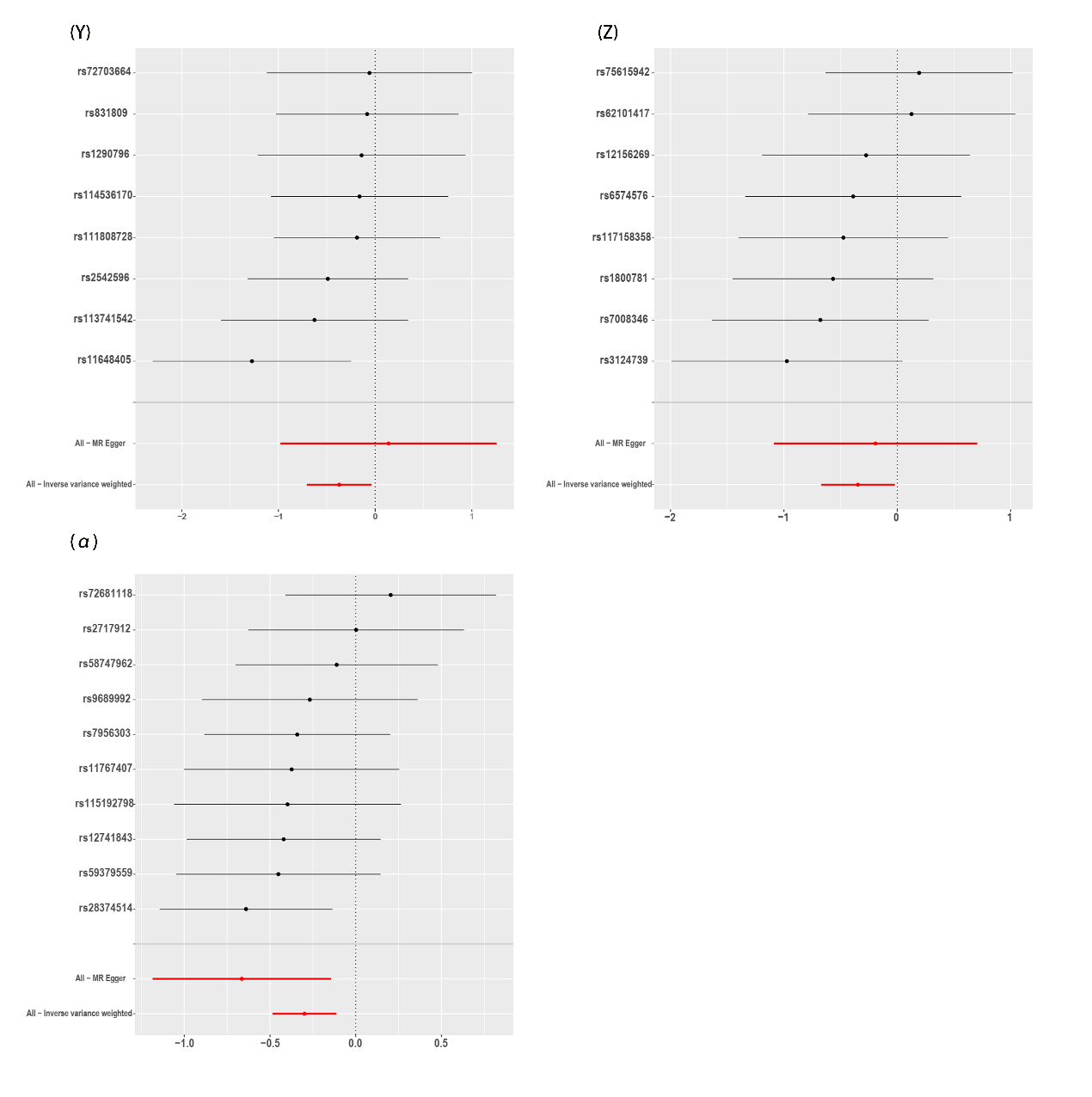


1. MR effect size for " Bifidobacteriaceae " on " Renal Cell Carcinoma ".
2. MR effect size for " Bifidobacterium infantis " on " Renal Cell Carcinoma ".
3. MR effect size for " Bifidobacterium " on " Renal Cell Carcinoma ".
4. MR effect size for " CAG-1031 " on " Renal Cell Carcinoma ".
5. MR effect size for " CAG-302 " on " Renal Cell Carcinoma ".
6. MR effect size for " CAG-345 sp000433315 " on " Renal Cell Carcinoma ".
7. MR effect size for " CAG-448 " on " Renal Cell Carcinoma ".
8. MR effect size for " CAG-590 sp000431135 " on " Renal Cell Carcinoma ".
9. MR effect size for " CAG-822 " on " Renal Cell Carcinoma ".
10. MR effect size for " CAG-826 " on " Renal Cell Carcinoma ".
11. MR effect size for " Desulfovibrionales " on " Renal Cell Carcinoma ".
12. MR effect size for " Desulfovibrio piger " on " Renal Cell Carcinoma ".
13. MR effect size for " Firmicutes A " on " Renal Cell Carcinoma ".
14. MR effect size for " Fusobacterium A " on " Renal Cell Carcinoma ".
15. MR effect size for " Halarcobacter " on " Renal Cell Carcinoma ".
16. MR effect size for " Hungatella sp900155545 " on " Renal Cell Carcinoma ".
17. MR effect size for " Megasphaera " on " Renal Cell Carcinoma ".
18. MR effect size for " Odoribacter laneus " on " Renal Cell Carcinoma ".
19. MR effect size for " Parachlamydiales " on " Renal Cell Carcinoma ".
20. MR effect size for " Photobacterium " on " Renal Cell Carcinoma ".
21. MR effect size for " Prevotellamassilia sp000437675 " on "Renal Cell Carcinoma".
22. MR effect size for " RUG147 sp900315495 " on " Renal Cell Carcinoma ".
23. MR effect size for " Terrisporobacter " on " Renal Cell Carcinoma ".
24. MR effect size for " Treponema D " on " Renal Cell Carcinoma ".
25. MR effect size for " UBA1409 " on " Renal Cell Carcinoma ".
26. MR effect size for " UBA737 " on " Renal Cell Carcinoma ".

(α) MR effect size for " Victivallis sp002998355 " on " Renal Cell Carcinoma ".
